# Clinical, Immunological, and Molecular Features of Typical and Atypical Severe Combined Immunodeficiency: Report of the Italian Primary Immunodeficiency Network

**DOI:** 10.3389/fimmu.2019.01908

**Published:** 2019-08-13

**Authors:** Emilia Cirillo, Caterina Cancrini, Chiara Azzari, Silvana Martino, Baldassarre Martire, Andrea Pession, Alberto Tommasini, Samuele Naviglio, Andrea Finocchi, Rita Consolini, Paolo Pierani, Irene D'Alba, Maria Caterina Putti, Antonio Marzollo, Giuliana Giardino, Rosaria Prencipe, Federica Esposito, Fiorentino Grasso, Alessia Scarselli, Gigliola Di Matteo, Enrico Attardi, Silvia Ricci, Davide Montin, Fernando Specchia, Federica Barzaghi, Maria Pia Cicalese, Giuseppe Quaremba, Vassilios Lougaris, Silvia Giliani, Franco Locatelli, Paolo Rossi, Alessandro Aiuti, Raffaele Badolato, Alessandro Plebani, Claudio Pignata

**Affiliations:** ^1^Pediatric Section, Department of Translational Medical Sciences, Federico II University, Naples, Italy; ^2^Department of System of Medicine University of Rome Tor Vergata, Rome, Italy; ^3^Unit of Immune and Infectious Disease, University Department of Pediatrics DPUO, Children's Hospital Bambino Gesù, Rome, Italy; ^4^Pediatric Immunology Unit, Anna Meyer Hospital, University of Florence, Florence, Italy; ^5^Department of Public Health and Pediatrics, Regina Margherita Children Hospital, University of Turin, Turin, Italy; ^6^Paediatric Hematology Oncology Unit, Policlinico-Giovanni XXII Hospital, University of Bari, Bari, Italy; ^7^Department of Pediatrics, S. Orsola-Malpighi Hospital, University of Bologna, Bologna, Italy; ^8^Pediatric Hematology Oncology, Institute for Maternal and Child Health IRCCS “Burlo Garofolo”, Trieste, Italy; ^9^Section of Pediatrics Immunology and Rheumatology, Department of Pediatrics, University of Pisa, Pisa, Italy; ^10^Division of Pediatric Hematology and Oncology, Ospedale G. Salesi, Ancona, Italy; ^11^Department of Child's and Woman's Health, Pediatric Oncology and Hematology, University of Padova, Padova, Italy; ^12^Pediatric Immunohematology and Bone Marrow Transplantation Unit, San Raffaele Telethon Institute for Gene Therapy (SR-TIGET), IRCCS San Raffaele Scientific Institute, Milan, Italy; ^13^Department of Advanced Biomedical Sciences, Federico II University, Naples, Italy; ^14^Department of Clinical and Experimental Sciences, Pediatrics Clinic and Institute for Molecular Medicine A. Nocivelli, University of Brescia, Brescia, Italy; ^15^A. Nocivelli Institute for Molecular Medicine, Department of Molecular and Translational Medicine, University of Brescia, and ASST Spedali Civili, Brescia, Italy; ^16^Department of Pediatric Hematology and Oncology, Bambino Gesù Children's Hospital, Rome, Italy

**Keywords:** primary immunodeficiencies, severe combined immunodeficiencies, atypical SCID, T-cell defects, lymphopenia, Omenn syndrome, maternal engraftment, next generation sequencing

## Abstract

Severe combined immunodeficiencies (SCIDs) are a group of inborn errors of the immune system, usually associated with severe or life-threatening infections. Due to the variability of clinical phenotypes, the diagnostic complexity and the heterogeneity of the genetic basis, they are often difficult to recognize, leading to a significant diagnostic delay (DD). Aim of this study is to define presenting signs and natural history of SCID in a large cohort of patients, prior to hematopoietic stem cell or gene therapies. To this purpose, we conducted a 30-year retro-prospective multicenter study within the Italian Primary Immunodeficiency Network. One hundred eleven patients, diagnosed as typical or atypical SCID according to the European Society for Immune Deficiencies criteria, were included. Patients were subsequently classified based on the genetic alteration, pathogenic mechanism and immunological classification. A positive relationship between the age at onset and the DD was found. SCID patients with later onset were identified only in the last decade of observation. Syndromic SCIDs represented 28% of the cohort. Eight percent of the subjects were diagnosed in Intensive Care Units. Fifty-three percent had an atypical phenotype and most of them exhibited a discordant genotype-immunophenotype. Pre-treatment mortality was higher in atypical and syndromic patients. Our study broadens the knowledge of clinical and laboratory manifestations and genotype/phenotype correlation in patients with SCID and may facilitate the diagnosis of both typical and atypical forms of the disease in countries where newborn screening programs have not yet been implemented.

## Introduction

Severe combined immunodeficiencies (SCIDs) are a large group of rare primary disorders due to defects in different genes involved in T- and often B- and NK- cell development or function, resulting in severe and sometimes life-threatening infections (1). The phenotypic complexity and heterogeneity of SCIDs may complicate the diagnosis thus frequently leading to a significant diagnostic delay (DD). To date, population-based newborn screening (NBS) represent the best strategy for the early identification of affected newborns prior to the onset of infections and other complications. The introduction of the NBS programs revealed that the prevalence of the disorder is much higher than the previously reported, accounting for 1:58,000 newborns, or even 1:7,300 live births, if other forms of T-cell lymphopenia are included (2, 3). However, it should be noted that NBS may fail to detect some CIDs, i.e., *ZAP70, ORAI1*, or MHC class II deficiencies, or SCIDs due to hypomorphic mutations, since in these cases thymic output may not be dramatically affected. Moreover, it is currently available in few Countries. For this reason, improving the awareness of the clinical and laboratory signs at presentation and the natural history may be particularly important to achieve a timely diagnosis (4–7).

Typically, SCID patients who have absent T cells can be further classified on the basis of the absence or presence of B and NK cells. To date, according to the International Union of Immunological Societies 17 different genes are recognized as responsible for T^−^B^+^ or T^−^B^−^ SCIDs. However, this number will progressively increase over the next few years (8–10), thanks to the availability of new high-throughput deep sequencing analysis. Thanks to this technology, different new SCID related genes, such as *EXTL3, BCL11B, DOCK2, LAT, ARPC1B* have been recently identified (11–15). The introduction of next generation sequencing in the clinical practice also allowed to identify new clinical phenotypes associated with well-known genetic defects, making more difficult the recognition of these conditions (16). SCID patients may often exhibit extra-immunological manifestations, which may even represent the dominant clinical phenotype at presentation, masking the underlying immunodeficiency. Hypomorphic mutations in some known SCID-causing genes may result in atypical presenting phenotypes, mainly characterized by immunodysregulation with a less remarkable increase of the susceptibility to infections. Eventually, a better definition of the clinical signs in patients with more severe forms compared to those with milder phenotypes, deserves careful attention for the implication in the overall management.

Finally, a better understanding of the clinical variability associated with the same gene defect, even within the same family, may help recognize patients (17) and unravel the complexity of the pathogenesis also in these well-established monogenic disorders.

The aim of the present study is to describe the clinical, immunologic and genetic features of a cohort of Italian patients affected with SCID identified within the activity of the Italian Primary Immunodeficiency Network (IPINET), with a special focus on the presenting signs and on the natural history prior to definitive therapeutic interventions.

## Materials and Methods

### Patient Source and Enrollment

This is a retrospective and prospective multicenter longitudinal clinical study involving patients with SCID identified by IPINET Centers. The primary aim of the study is to better define the presenting signs and the clinical history of these complex disorders prior to hematopoietic stem cell (HSCT) or gene therapies (GT).

Patients who received a diagnosis of typical or atypical SCID between January 1, 1986 and December 31, 2017, were included in the study, after written informed consent had been obtained from the participants and parents/legal guardians. Patients identified through NBS program, currently available only in Tuscany and Campania, have been excluded. Eligibility was verified by the principal investigator on the basis of inclusion and exclusion criteria. Typical SCID was defined according to the European Society for Immunodeficiencies (ESID) criteria in patients with: (1) mutation(s) in a gene associated with a typical SCID phenotype; or, (2) presentation with severe or opportunistic infections, persistent diarrhea and failure to thrive, in the presence of low (300**/**μl) or absent CD3^+^ or CD4^+^ or CD8^+^ T cells, reduced naive CD4^+^ (CD3^+^CD4^+^CD45RA^+^) and/or CD8^+^ (CD3^+^CD8^+^CD45RA^+^) T cells, elevated γδ T cells, absence of proliferative responses to mitogens, defined as proliferative response to phytohemagglutinin (PHA) lower than 10% of the control subject; or (3) T cells of maternal origin present. However, differently from ESID criteria, we also included patients with onset after the first year of life when the other criteria were fulfilled. Atypical SCIDs were characterized by CD3^+^ > 300 cells/μl and reduced, but detectable, proliferative response to PHA (> 10 < 30% of the control). In order to avoid the inclusion of patients with milder forms, as combined immunodeficiency (CID), only atypical SCID younger than 2 years of age, were included. The following reference values were used to characterize the immune phenotype: B^+^ (B-cell count > 400 mm^3^), B^low^ (50 to 400 mm^3^), or B^−^ (< 50 mm^3^) and natural killer (NK)^+^ (NK-cell count > 100 mm^3^), NK^low^ (40 to 100 mm^3^), or NK^−^ (< 40 mm^3^) according to Pai et al. (10) Omenn syndrome (OS) was defined in presence of desquamating erythroderma, absence of maternal engraftment (ME), absent or low (< 30% of normal) T-cell proliferation, low naïve cells, and at least 1 of the following supportive criteria: hepato-splenomegaly, lymphadenopathy, failure to thrive, chronic diarrhea, increased IgE level or absolute eosinophil count, and peripheral expansion of self-reactive oligoclonal T cells (18). Different techniques were used to detect ME, including cytogenetics (XX/XY chimerism assay by FISH in male subjects, *n* = 3), restriction fragment length polymorphism analysis (RFLP, *n* = 3) in patients diagnosed before 2006, short tandem repeat analysis (STR, *n* = 34) or HLA high resolution typing (*n* = 13) for patients diagnosed in the third decade of observation. In all the cases, the analysis was performed on PBMCs. ME was considered unlikely if CD3+ T cells were undetectable (< 100 cells/mm^3^) or very low (> 100 and < 300 cells/mm^3^) in patients with no clinical signs of ME and no analysis was performed in these cases. In addition, patients with known genetic defect were grouped according to the main pathway implicated as follows: Metabolic Disorders (MD), due to inherited disorders of the purine metabolism, such as Adenosine Deaminase (*ADA*) deficiency; Cytokine Signaling Defects (CSD), whose prototype was X-Linked SCID due to mutations in the interleukin 2 receptor gamma (*IL2RG)* gene, a component of several cytokine receptors; V(D)J Recombination Disorders (VDJ), mainly caused by mutations in the recombination activating genes (*RAG*) *1* and *2*; DNA Repair Defects (DNA-RD), due to alterations in genes involved in preserving genomic stability; Thymic Disorders (TD), due to abnormalities of the stromal component of the thymus. Patients with secondary immunodeficiencies, with other well-defined PIDs such as Ataxia-Telangiectasia, Hyper-IgE syndrome, Wiskott-Aldrich syndrome, Dyskeratosis congenital and/or incomplete diagnostic criteria were excluded. Age at Onset (AO) was defined as the age at the first documented SCID-related symptoms, Age at Diagnosis (AD) as the age at diagnosis of SCID, while Diagnostic Delay (DD) was defined as the difference between the two values. Patients were defined syndromic if they showed at least 2 non-immunological symptoms such as: peculiar facial dysmorphic features, microcephaly, cognitive impairment, ectodermal abnormalities, renal defects, or gut abnormalities. The number of signs was calculated taking into account the number of events indicated by the Referents of the participating Centers for each category. Categories analyzed were: infectious diseases, including bacterial, viral, or opportunistic infections of respiratory tract, central nervous system (CNS), skin and lymph node, gastrointestinal and muscolo-skeletal system, systemic sepsis; gastrointestinal manifestations, including chronic diarrhea, failure to thrive; eczema; manifestation due to immune dysregulation as autoimmune cytopenia, granuloma, ectodermal disorders, vasculitis, thyroiditis; hematological disorders including hemophagocytic lymphohistiocytosis, systemic or localized lymphadenopathy, lymphoproliferative disorders. Organomegaly, ME and OS were also taken in consideration. We counted only once each clinical sign within the category. The study, conducted according to the World Medical Association Declaration of Helsinki ethical principle, was approved by the Coordinator Center Ethics Committee (Federico II Ethics Committee, registration number 61/17) and further approved by local Ethics Committees of all participating groups. Complete immunological characterization was available for 99 (89%) patients. Genetic analyses were performed in 88 (79%) patients, since the molecular characterization was very limited in the 80's and only recently deep sequencing technologies have been widely introduced into the clinical practice. All laboratory results, including complete blood count, were analyzed with reference to age-related normal ranges.

### Statistical Analysis

Data were shown as mean ± SD, median and range, or frequencies (number of cases) and percentages as appropriate. Student's *t*-test was used to compare means for continuous variables. Chi-square test or Fisher's exact test were used for categorical variables. Comparisons between groups were performed using the non-parametric Mann-Whitney test for quantitative variables. All *P*-values are two-sided and values < 0.05 were considered significant. Furthermore, we performed a hypothesis-free multivariate analysis, using factor analysis. This method divides total data variability into components to explain the largest amount of variability in the data. A scree plot was used to select the appropriate number of components for evaluation. A factor loading size of 0.7 or greater indicates that a variable was associated with that component. Varimax rotation was used to enable a clearer interpretation of the results. Variables with a highly skewed distribution were analyzed on the log scale. The calculations were performed using IBM SPSS Statistics, v.20.0 software (IBM Corp. Armonk, NY).

## Results

### Demographic Features

**Figure 1:**
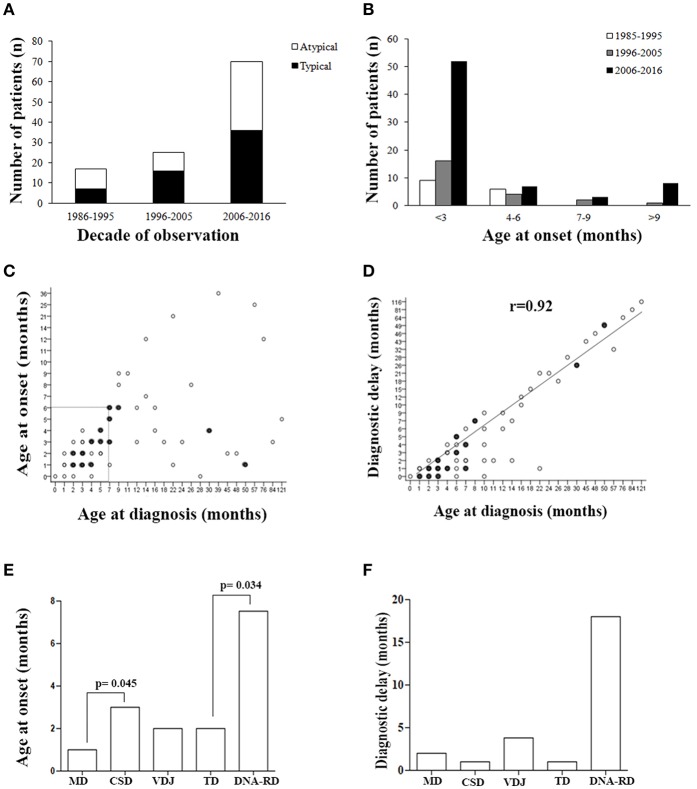
Patients' demographics. **(A)** Number of typical or atypical SCID patients diagnosed during the overall study period. **(B)** Histogram distribution of patients grouped by age at onset. **(C)** Correlation between age at onset and age at diagnosis, and **(D)** between age at diagnosis and diagnostic delay. Filled circles indicate groups of SCID patients (between 2 and 5) with same values. **(E)** Age at onset and **(F)** diagnostic delay (median) for the various pathogenic group of SCID. MD, Metabolic Disorders; CSD, Cytokine Signaling Disorders; VDJ, VDJ abnormalities; TD, Thymic Disorders; DNA-RD, DNA Repair Disorders.

Table 1 summarizes the demographic data. The cohort included 111 patients (56.7% males). Most of the patients (*n* = 86) were of Italian origin, while the remaining 22.6% (*n* = 25) of the subjects were of a different geographic origin. Ninety-four percent of them were diagnosed based on the clinical history and presenting signs. Prenatal or pre-symptomatic investigations in families with other affected babies, led to the diagnosis of SCID in 1 (0.9%) and 5 (4.5%) cases, respectively. A familial history suggestive of SCID, including early infant deaths or other PIDs signs was reported in 23.3% of cases. Thirty-three percent of the infants were referred to an IPINET Center before the 3rd month of life, while 22.5% of the patients were referred after 1 year of life. A significant proportion of patients (28%) had a syndromic phenotype with more than two non-immunological features. Most of the patients (62.7%) were diagnosed in the last decade (2006–2016), while only the 15.5% were diagnosed in the first decade (1986–1995) (Figure 1A). The proportion of typical and atypical SCIDs was similar across the entire period of observation.

**Table 1:** Demographic features of the SCID cohort.

	**Total (*****n*** **=** **111)**		**Non-syndromic (*****n*** **=** **80)**		**Syndromic (*****n*** **=** **31)**	
	***N* total**	**Percent cases**	***N* total**	**Percent cases**	***N* total**	**Percent cases**
**Gender**						
Male	63	56.7	52	65	11	35.5
Female	48	43.3	28	35	20	64.5
**Country of origin**						
Italy	86	77.4	58	72.5	28	90.3
North-Africa	5	4.5	3	3.7	2	6.4
Tunisia	1	0.9	1	1.25	0	0
Morocco	2	1.8	1	1.25	1	3.2
Senegal	1	0.9	1	1.25	0	0
Lybia	1	0.9	0	0	1	3.2
USA/South America	3	2.7	2	2.5	1	3.2
Asia	7	6.3	7	8.7	0	0
Saudi Arabia	1	0.9				
Iran	1	0.9				
Lebanon	2	1.8				
India	1	0.9				
Turkey	1	0.9				
Pakistan	1	0.9				
Eastern Europe	8	7.2	8	7.2	0	0
Romania	5	4.5				
Serbia	1	0.9				
Macedonia	2	1.8				
Northern Europe	2	1.8	2	2.6	0	0
Netherland	1	0.9				
Belgium	1	0.9				
**Positive family history**						
SCID	16	14.9	9	11.25	7	22.6
Other PIDs	3	2.8	1	1.25	2	6.4
Early infant death	6	5.6	2	2.5	4	13

As shown in Figure 1B, a considerable increase of early diagnoses (< 3 months of age) was noted in the last decade of observation and almost all late onset forms (> 9 months) were diagnosed in the last decade of observation. Within the early onset group (AO ≤ 9 months), the median AO of the disease was 2 months (range 0–9 months), while in the late onset group (AO > 9 months) it was 13 months (range 10–36 months) (*P* <0.0001). Sixty-nine out of 111 pts (62%), whose AO was within the first 6 month of life, received a diagnosis by the 7th month of life (Figure 1C). As shown in the Figure 1, there was a significant direct correlation between AD and diagnostic delay (DD) (Figure 1D). AO was lower in patients with MD compared to CSD (*p* = 0.045) and in those with TD compared to DNA-RD (*p* = 0.034) (Figure 1E). DD was higher in patients with DNA-RD (median 18 months), even though the difference was not statistically significant (Figure 1F).

### Characteristics of the Infants at Diagnosis

Table 2 summarizes the clinical features of the cohort. Almost all the subjects (95.5%) were symptomatic at diagnosis. In particular, 91 patients had 4 or more clinical signs at the diagnosis, including severe or persistent infections and/or immunedysregulation, while 13 patients had more than 10 signs. Most of the patients with atypical SCID had more than 10 signs (Supplemental Figure 1). Infections were documented at diagnosis in 94 of the 111 children (85%). The most common manifestations of infections were: lobar and/or interstitial pneumonia (54%), otitis (18%), bronchiolitis (16%), and sepsis (9%). Thirteen percent of pneumonia were interstitial pneumonia due to *Pneumocystis Jiroveci* confirmed by positive microscopy, while 22% were of undetermined origin. Meningitis was documented only in 3 patients (3%). Other infectious manifestations included severe/systemic viral infections (28%), and were due to CMV (16%), EBV (6%), HSV (4.5%), and adenovirus (1%). Other opportunistic or BCG/mycobacterial infections were documented in 22 and 5% of the patients, respectively. Five patients who had BCG infection came from Countries where BCG vaccine is administered. Interestingly, 9 previously healthy children presented with a severe infection rapidly worsening and requiring admission to intensive care unit (ICU) (8.1%). OS was found in 19 subjects (18%). Thirty-four percent of the 53 patients analyzed were positive for ME. In 3 of the 18 subjects with ME, a fully expressed Omenn-like disease, characterized by erythrodermia or severe eczema, alopecia, hepatosplenomegaly, and eosinophilia, was observed.

**Table 2:** Clinical features of patients at diagnosis.

**Feature**	**Overall (%)**	**Non-syndromic (%)**	**Syndromic (%)**	***P*-value**
	**No = 111**	**No = 80**	**No = 31**	
Infections (N)	94 (85)	65 (76)	29 (93.5)	0.147
Major respiratory tract infections	75 (67)	51 (64)	24 (77)	0.356
Pneumonia	65 (54)	44 (55)	21 (68)	0.389
Lobar	26 (23)	20 (25)	6 (19)	0.528
Interstitial pneumopathy	37 (33)	23 (29)	14 (45)	0.099
Lobar+Interstitial	2 (2)	1 (1)	1 (3)	0.482
*P. Jiroveci*	14 (13)	9 (11)	5 (16)	0.541
Unspecified	25 (22)	15 (19)	10 (32)	0.209
Bronchiolitis	18 (16)	14 (17.5)	4 (13)	0.580
Bronchiectasis	3 (3)	2 (2.5)	1 (3)	0.832
Other severe bacterial infections	19 (17)	9 (11)	10 (32)	**0.023**
Sepsis	11 (9)	6 (7.5)	5 (16)	0.291
Meningitis	3 (3)	0 (0)	3 (10)	**0.023**
Skin and lymph node abscesses	4 (4)	3 (4)	1 (3)	1.00
Omphalitis	1 (1)	0 (0)	1 (3)	0.290
Otitis	20 (18)	14 (17.5)	6 (3)	1.00
Systemic viral infections	31 (28)	20 (25)	11 (35.5)	0.356
CMV	18 (16)	12 (15)	6 (19)	0.776
EBV	7 (6)	4 (5)	3 (10)	0.411
HSV1/2	5 (4.5)	3 (4)	2 (6)	0.626
Adenovirus	1 (1)	1 (1)	0 (0)	1.00
Opportunistic infections	25 (22)	17 (21)	8 (26)	0.802
Candidiasis	19 (17)	12 (15)	7 (23)	0.414
Disseminated fungal infection	6 (5)	5 (6)	1 (3)	0.670
BCG infection	5 (4.5)	4 (5)	1 (3)	1.00
***Mycobacterium tuberculosis***	1 (1)	0 (0)	1 (3)	0.290
OS	19 (18)	14 (17)	5 (16)	1.00
ME^*^	15 (28)	12 (33)	3 (18)	0.236
Omenn-like +ME^*^	3 (6)	3 (8)	0 (0)	0.293
Gastrointestinal features	54 (49)	35 (44)	19 (61)	0.201
Chronic diarrhea	27 (24)	21 (26)	6 (19)	0.466
Failure to thrive	39 (35)	21 (26)	18 (58)	**0.004**
Villous atrophy	6 (5)	1 (1)	5 (16)	**0.008**
Protein loss entheropathy	6 (5)	2 (2.5)	4 (13)	0.057
Eczema	27 (24)	17 (21)	10 (32)	0.467
Transient or treatable	4 (4)	3 (4)	1 (3)	1.00
Untreatable	10 (9)	8 (10)	2 (6)	0.720
Erythrodermia	13 (12)	6 (7.5)	7 (23)	**0.050**
Immune dysregulation	25 (22)	15 (19)	13 (42)	**0.028**
Autoimmune cytopenia	13 (13)	8 (10)	6 (19)	0.193
Granuloma	3 (2)	1 (1)	2 (6)	0.201
Alopecia/ectodermal dystrophy	5()	1(1)	4 (13)	0.204
Vasculitis	2 (2)	2 (2.5)	0 (0)	1.00
Thyroiditis	2 (2)	1 (1)	1 (3)	0.497
Hematological disorders	17 (15)	12 (15)	5 (16)	1.00
HLH	4 (4)	3 (4)	1 (3)	1.00
Lymphadenopathy	13 (12)	8 (10)	5 (16)	0.516
Lymphoproliferative disorders	2 (2)	1 (1)	1 (3)	0.497
Organomegaly	54 (48.5)	36 (45)	18 (58)	0.395
Liver enlargement	31 (28)	22 (27.5)	9 (29)	0 1.00
Spleen enlargement	23 (20.5)	14 (17)	9 (29)	0.301
Intensive care unit admission	9 (8)	7 (9)	2 (6)	0.690

CMV, cytomegalovirus; EBV, epstein barr virus; HSV, Herpes simplex virus; BCG, Bacillus Calmette Guerin; OS, Omenn syndrome; ME, Maternal Engraftment; HLH, Haemophagocytic Lymphohistiocytosis.

Bold indicates statistical significance (P < 0.05).

* Frequency was calculated as percentage of patients tested for ME (n = 53; syndromic n = 17).

Gastrointestinal disorders were detected in 49% of the children, failure to thrive being the most frequent clinical manifestation (35%). Chronic diarrhea was reported in 24% of cases. Severe protein-losing enteropathy and villous atrophy were reported in 6 patients (5%).

The most common dermatological alteration was eczema, present in 24% of the patients. Four percent showed a localized easy-to-treat dermatitis, 9% a moderate-diffuse form and 12% erythroderma or a severe, diffuse, difficult-to-treat eczema. Other dermatological disorders included alopecia and/or ectodermal abnormalities, as detailed in the Table 2.

Overall, 25 patients (22%) showed signs of immune dysregulation at the initial clinical and functional evaluation. Among these, 13 had autoimmune cytopenia, 3 presented with granulomatous disease, and 2 had thyroiditis or vasculitis.

Hematological disorders were reported in 17 patients (15%), including HLH in 4 cases, localized or systemic lymphadenopathy in 13, and lymphoproliferative disorders in 2 of them. Failure to thrive, villous atrophy, meningitis or other severe bacterial infections, immunedysregulation and erythroderma were significantly more frequent in syndromic vs. non syndromic patients and in atypical vs. typical SCID groups, as shown in the Table 2 and in Supplemental Table S1. On the contrary, severe systemic viral infections, OS, eczema and autoimmune cytopenia, more frequent in atypical SCIDs (Supplemental Table S1).

As detailed in Table 3, extra-immunological signs were identified in 31 subjects, and mainly included birth defects, such as musculoskeletal or genitourinary tract malformation, or neurological manifestations. Neurological manifestations included both sensory or motor deficits, and were unlikely to be caused by infections, since only two patients suffered from a severe invasive infection of CNS. EBV and CMV-DNA levels were evaluated in peripheral blood through real-time polymerase chain reaction (PCR) in 15 out of 19 subjects. Five of them had a systemic CMV infection (CMV viremia). The age at presentation suggested a postnatal acquisition of CMV rather than congenital infection, even though, in most of the cases, we couldn't rule out a congenital infection, since samples obtained within 3 weeks of age were not available. In one case, PCR analysis of dried blood samples on the Guthrie card was performed to exclude a congenital infection. In 2 subjects, PCR on peripheral blood was positive for EBV or adenovirus, respectively, but cerebrospinal fluid (CSF) liquor examination was negative. Eleven patients had dysmorphic features and one had situs viscerum inversus.

**Table 3:** Extra-immunological features of the SCID cohort.

**Features**	***n*. of subjects (%)**
**Overall**	31 (28)
**Birth defects**	
Facial dysmorphism	11 (35)
Skeletal anomalies	7 (22.5)
Genitourinary anomalies	4 (13)
Gastrointestinal anomalies	2 (6)
Situs Viscerum Inversus	1 (3)
**Congenital heart disease**	
Conotruncal defects	1 (3)
Septal defects	3 (10)
**Neurological disorders**	
Microcephaly	7 (23)
Unexplained cerebral atrophy	1 (3)
Sensorineural hearing loss	1 (3)
Developmental delay	9 (29)
Epilepsy	1 (3)
Hypotonia	3 (10)
Flaccid tetraparesis	1 (3)
Palpebral ptosis	1 (3)
Other	2 (6)

Twenty-one percent of the subjects had anemia, which was severe (Hb < 8 g/l) in 40% of them (9/23). Neutropenia was observed in 16.5% of the patients (*n* = 15), with a mean absolute neutrophil count of 646.8 ± 401.6 cells/mm^3^ (range 0–1447 cell/mm^3^). Bilinear (neutropenia + anemia) or trilinear cytopenias (neutropenia + anemia + thrombocytopenia) were detected in 4 and 2 patients, respectively. More than half of subjects had eosinophilia and in 60% of the cases, it was not associated with OS. However, eosinophilia was more severe (> 2,000 cell/mm^3^) in patients with OS/ME. Thrombocytosis was observed in 48.5% of the patients (range 452.000–1.092.000 cells/mm3). Multivariate analysis showed an interdependency between thrombocytosis, infection and inflammatory skin disorders (eczema) (data not shown). One patient with *IL2RG* defect developed a lymphoproliferative disorder not EBV-related, while a patient carrying *DLREC1A* biallelic genetic alterations developed an EBV-related large B-cell lymphoma of nasopahrynx. The same subject also developed a primary cutaneous CD8 + acral T-cell lymphoma.

We performed Fisher's exact test to compare the prevalence of all phenotypic variables in the subgroups of the cohort. Non lymphopenic patients showed a higher prevalence of hematological disorders compared to lymphopenic patients (*p* = 0.046). DD was not different between these 2 groups. Bronchiectasis were more frequent in patients with early onset (AO ≤ 9 months) compared to late onset forms (*p* = 0.011).

**Figure 2:**
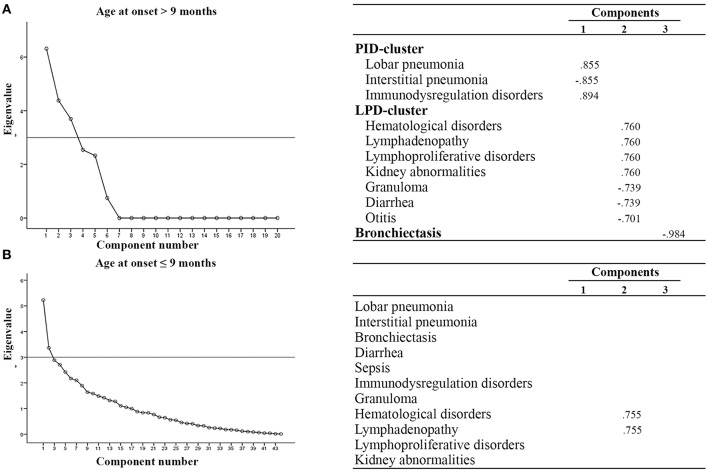
Multivariate analysis in SCID patients according to age at onset. **(A)** AO > 9 mo, **(B)** AO ≤ 9 mo. All components with eigenvalues under 3 were dropped. Factor loadings in the analysis are illustrated in the right part of the panel. Clusters of variables positively correlated are indicated. LPD-cluster, lymphoproliferative disorder-cluster; IDP-cluster, Immuno-dysregulation and pulmonary infection-cluster.

We next performed multivariate analysis to obtain information about the interdependency between the observed variables. In particular, patients were stratified according to age at onset (before or after 9 months of age), outcome (alive or dead), diagnostic delay (more or less than 3 months), syndromic or non syndromic patients and immunological features (lymphopenic or not lymphopenic, typical or atypical). With an eigenvalue > 3, varimax rotation yielded a 3-factor model where a few variables are correlated one to each other. As shown in Figure 2, we found that patients with AO > 9 months had additional clusters of disorders, as compared to patients with AO ≤ 9 months, assuming as eigenvalue cut-off of 3: the first characterized by benign/malignant lymphoproliferation, hematological disorders and granuloma (LPD-cluster); the second characterized by immunedysregulation associated with pulmonary infections (IDP-cluster). The analysis did not reveal any significant difference in phenotypic presentation clusters in patients stratified according to the other parameters.

### Genotype Characterization

A genetic diagnosis was obtained in 70% (77/111) of all the patients and in 87.5% (77/88) of the children who underwent molecular investigation. Most of cases (71.5%) were studied through a traditional candidate gene approach using Sanger direct sequencing. Genes were selected based on the immunophenotypic classification, the gender, clinical features and inheritance pattern. In the 28.5% of the cases, a genetic diagnosis was obtained using high-throughput sequencing (HTS). This technique was introduced in most of the Centers in the last decade. In 2 patients with a complete DiGeorge (cDGS) clinical phenotype (2.5%, 2/77) CGH-array analysis revealed a typical 22q11.2 deletion and a 14q deletion, respectively.

**Figure 3:**
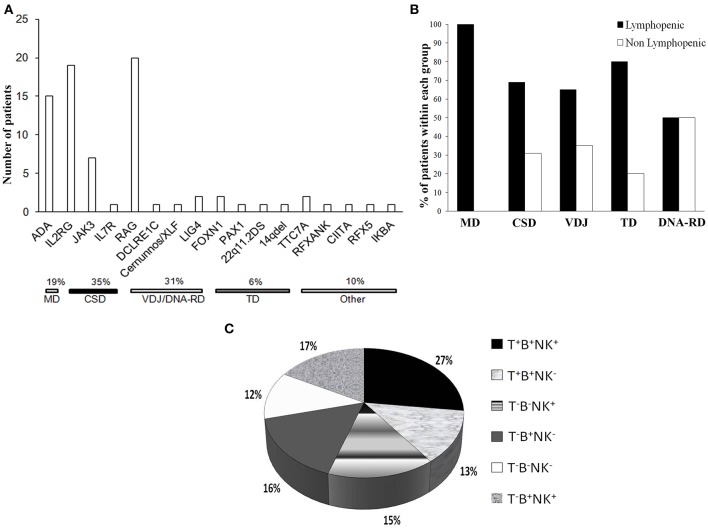
Genetic causes and immunophenotype of the cohort. **(A)** Histogram distribution of the genetic defects underlying SCID. The pathogenic mechanisms implicated are indicated. *ADA*, adenosine deaminase; *IL2RG*, interleukin 2 receptor gamma; *JAK3*, Janus kinase 3; *IL7R*, interleukin 2 receptor; *RAG*, recombinase-activating genes; *LIG4*, DNA Ligase IV; *FOXN1*, Forkhead Box N1; *PAX1*, Paired Box Gene 1; *TTC7A*, Tetratricopeptide Repeat Domain-Containing Protein 7A; *RFXANK*, Regulatory Factor X, Ankyrin Repeat-Containing; CIITA, Class II Transactivator; *RFX5*, Regulatory Factor X; *IKBA*, NFKB inhibitor alpha. **(B)** Proportion of lymphopenic or non lymphopenic patients according to the pathogenic group. **(C)** Distribution of the cohort according the immunophenotype, expressed as percentage values.

CSD was the most frequent genetic cause, accounting for 35% of the cases (Figure 3A), while VDJ abnormalities and DNA-RD accounted for the 31% of the cases. Twenty patients (26%) had alterations in *RAG1/2* genes and 4 (5%) had defects in the DNA repair machinery (*DCLRE1C, Cernunnos/XLF, LIG4*). *ADA* deficiency was diagnosed in 15 subjects (7 of them being of other geographic origin). Six percent of the patients had a SCID caused by a primary thymic developmental disorder (*PAX1, FOXN1*, cDGS). Rare genetic causes, as *PAX1* and *TTC7A* deficiencies were identified through HTS.

Within the group of SCID patients who received a molecular characterization, the rate of a positive molecular diagnosis was 86% among patients of Italian origin (57 out of 66 patients) and 91% (20 out of 22 patients) among patients of different geographic areas.

### Immunological Characterization at Diagnosis

Most of the patients (75%) were lymphopenic at the diagnosis, while 24 patients (24.2%) had normal or even increased lymphocyte counts, as compared to age-matched reference values. Nine out of 19 OS patients were lymphopenic while 7 had increased lymphocyte counts. Most of the non lymphopenic patients had CSD or VDJ disorders (31 or 35%, respectively) and 50% of the subjects with DNA-RDs had a normal lymphocyte count. None of MD patients had a near normal lymphocyte count (Figure 3B). Three non lymphopenic patients had ME.

As shown in the Figure 3C, the majority of the patients (60%) had a T^−^ form, 50% of whom being B^−^. Within the T^−^B^+^ subgroup, NK cells were absent in approximately 50% of the patients. Among patients with T^−^B^−^ SCID, 41% were also NK^−^ negative.

We next evaluated the immunophenotype in the group of 35 patients with atypical SCID of known genetic basis. All patients but 3 (2 with *FOXN1* and 1 with *RFXANK* deficiency*)* had an atypical immunophenotype, as compared to what expected on the basis of the genotype. As shown in Table 4, the majority of atypical phenotypes were noted in 12 patients in the subgroup of the cytokine signaling defects (*IL2RG* or *JAK3*). In this group, the presence of T cells was potentially explained by OS/ME only in 2 cases. In addition, all the 8 *IL2RG* deficient patients without clinical manifestations of OS/ME also had normal/elevated NK cells. Interestingly, the mutation in the *IL2RG* gene was hypomorphic in one patient, while the remaining subjects carried severe mutations. Only 5 patients (11%) among typical SCID had an unpredicted phenotype as compared to genotype. More detailed site-specific mutations, with hypomorphic or severe effect, in the group of atypical subjects are listed in Table 5.

**Table 4:** SCID patients with known gene defect and discordant immunophenotype.

**Gene defect**	**Expected phenotype**	**Observed phenotype**	**No**.	**% of the genotype subgroup**	**OS/ME**
Typical			5		
*IL2RG*	T^−^B^+^NK^−^	T^−^B^+^NK^+^	1	12.5	0/0
		T^−^B^low^NK^+^	1	12.5	0/0
*RAG1/2*	T^−^B^+^NK^+^	T^−^B^−^NK^+^	1	14	0/1
*ADA*	T^−^B^−^NK^−^	T^−^B^+^NK^+^	1	8	0/0
MHC class II-D^*^	T^+^B^+^NK^+^	T^−^B^low^NK^+^	1	100	0/0
Atypical			31		
*IL2RG*	T^−^B^+^NK^−^	T^low^B^+^NK^+^	6	31.5	0/1
		T^+^B^+^NK^+^	3	16	1/1
		T^low^B^+^NK^low^	1	5	0/0
*JAK3*	T^−^B^+^NK^−^	T^low^B^+^NK^−^	1	14	0/0
		T^low^B^+^NK^+^	1	14	0/0
*RAG1/2*	T^−^B^+^NK^+^	T^+^B^−^NK^+^	7	35	3/0
		T^low^B^+^NK^−^	2	10	1/1
		T^+^B^+^NK^+^	3	15	1/0
		T^low^B^−^NK^−^	1	5	1/0
MHC class II-D^*^	T^+^B^+^NK^+^	T^low^B^+^NK^+^	3	75	0/0
*IKBA*	T^+^B^+^NK^+^	T^+^B^+^NK^−^	1	100	0/0
*Cernunnos*	T^−^B^−^NK^+^	T^low^B^−^NK^+^	1	100	1/0
*RFX5*	T^+^B^+^NK^+^	T^low^B^+^NK^?^	1	100	0/0
*DCLER1C*	T^−^B^−^NK^+^	T^+^B^low^NK^−^	1	100	0/0
*LIG4*	T^−^B^−^NK^+^	T^low^B^low^NK^+^	1	50	0/0
*CIITA*	T^−^B^+^NK^+^	T^+^B^+^NK^+^	1	100	0/0
*Del14*	T^−^B^+^NK^+^	T^low^B^−^NK^+^	1	100	0/0

* *Major histocompatibility complex (MHC) class II Deficiency. OS, Omenn syndrome; ME, Maternal Engraftment*.

**Table 5:** Genetic mutations in atypical SCID due to cytokine signaling disorders.

**Gene**	**ID Pt**	**Nucleotide change**	**Status**	**Predicted change**	**Hypomorphic**	**Severe**
*IL2RG*	9	c.676C>T	Hemyzygous	R226C	-	+
	24^a^	c.202G>A	Hemyzygous	E68K	+	-
	30	c.548T>C	Hemyzygous	L183C	-	+
	68	c.334A>C	Hemyzygous	T112P	+	-
	73	c.684C>T	Hemyzygous	R224W	-	+
	79	c.684C>T	Hemyzygous	R224W	-	+
	80	c.485T>G	Hemyzygous	L162R	-	+
	83^b^	c.981_1001del	Hemyzygous	R328E334del	-	+
	90	c.854G>A	Hemyzygous	Aberrant splice	-	+
	105	c.741insG	Hemyzygous	S248fs	-	+
*JAK3*	11	c.1208G>A	Homozygous	R403H	+	-
	111	c.1796T>G	Compound	V599G	+	-
		c.2125T>A	Heterozygous	W709R	+	-

a Omenn-like syndrome + Maternal Engraftment.

b *Maternal Engraftment*.

OS or ME were identified in 7 out of 13 patients with *RAG1/2* defects, and in an individual patient carrying a defect in Cernunnos/XLF.

We, finally, performed an intrafamilial comparison of the immunological and clinical phenotype in 4 families with 2 affected siblings. As shown in Table 6, in 3 pedigrees we observed a discordant immunological phenotype. In particular, differently from their siblings, patients A1 and B2 had low T cells. Furthermore, NK cells were low in A1, but normal in his brother. Patient C1 had a T^low^ SCID, with reduced but detectable CD3^+^ and CD8^+^ cells and a severe CD4^+^ lymphocytopenia, while his brother had a T^−^SCID with severe reduction of both CD4^+^ and CD8^+^ cells. Of note, ME, which could have explained the variability in the CD8 count, was ruled out in patient C1 by STR analysis. Patient A1 developed severe protracted diarrhea and hypoproteinemia at 1 month of age followed by bronchiolitis at 1.5 month of age. He was hospitalized because of failure to thrive at the age of 4 months. His younger brother (patient A2) was diagnosed with SCID at the age of 3.5 months because of a positive family history and clinical manifestations of OS (erythroderma, lymphadenopathy, spleen enlargement, eosinophilia, increased number of activated T-cells, reduced proliferative response to mitogen). RFLP ruled out ME. Patient C1 developed *Pneumocistis Jiroveci* pneumonia at the age of 3 months and died of severe respiratory failure and pulmonary arterial hypertension, while his asymptomatic brother was diagnosed because of the positive familial history. The clinical phenotype of the two of *FOXN1* siblings has been previously reported (19). The outcome was also discordant in 2 pairs, even though this was likely due to difference in DD rather than to the different immunological phenotype.

**Table 6:** Clinical and immunological features of SCID siblings.

**Patient ID**	**AO**	**AD**	**DD**	**Gene**	**Onset sign**	**OS**	**ALC cells/μL**	**CD3^**+**^ cells/μL**	**CD4^**+**^ cells/μL**	**CD8^**+**^ cells/μL**	**CD19^**+**^ cells/μL**	**CD56^**+**^ cells/μL**	**Immunophenotype**	**Outcome**	**Cause of deaths**
A1	1	4	3	Unknown	Chronic diarrhea	-	1100^a^	972^a^	720^a^	306^a^	18^a^	616^a^	T^low^B^−^NK^+^	dead	Systemic viral infection
A2	0.5	3.5	3	Unknown	Erythroderma	+	22255	9258	4206	5786	89^a^	3116	T^+^B^low^NK^+^	dead	OS
B1	2	6	4	*FOXN1*	Eczema	+	5392	2006	1065	439	3950	1442	T^+^B^+^NK^+^	dead	Pneumonia
B2	1	1	0	*FOXN1*	Eczema	+	2688	753^a^	645^a^	215^a^	962	650	T^low^B^+^NK^+^	alive	
C1	3	8	5	*IL2RG*	*P. Jiroveci* pneumonia	-	5528	719^a^	55^a^	685^a^	4765	NA	T^low^B^+^	dead	PH
C2	12	12	0	*IL2RG*	Asymptomatic	-	931^a^	19^a^	2^a^	9^a^	847	7.4^a^	T^−^B^+^NK^−^	alive	
D1	3	5	2	*JAK3*	Chronic diarrhea	-	1824^a^	62^a^	40^a^	18^a^	1621	11^a^	T^−^B^+^NK^−^	alive	
D2	2	2	0	*JAK3*	Asymptomatic	-	3926	12^a^	0^a^	1^a^	3299	10^a^	T^−^B^+^NK^−^	alive	

a *< normal age range value; ALC, Absolute Lymphocyte count; NA, not available; OS, Omenn syndrome; PH, pulmonary arterial hypertension*.

### Pre-treatment Mortality

The mortality rate before any definitive treatment (HSCT or GT) was 14.4% (16 cases). Seven patients died of severe infections, while OS or HLH were the cause of death in 3 further subjects. Multi-organ failure, pulmonary hypertension or severe electrolyte disorders were the causes of death in the remaining 6 patients.

Pre-treatment mortality was higher in patients with atypical vs. typical SCIDs (13/53 vs. 3/46*, p* = 0.0263) and in syndromic vs. non-syndromic SCID patients (9/31, 29 vs. 7/80, 9%, respectively; *p* = 0.0188). All the subjects who died before definitive treatment had an AO <underline<> 9 months. No association was found between DD > 3 months (*n* = 5) and pre-treatment mortality. We performed a multivariate analysis to evaluate the phenotypic cluster of signs in patients who died before treatment, a multivariate analysis. In atypical patients with poor prognosis, the clinical phenotype was mainly characterized by 4 clusters of symptoms, represented by a skin-blood cluster, 2 PID-clusters, and growth-development cluster (Supplemental Figure S2). The analysis was not performed in typical SCID due to the limited number of subjects.

## Discussion

The main aim of the IPINET study was to better define the natural history of different forms of SCIDs in order to improve the clinical management and increase the awareness of physicians on these often under recognized disorders. In this regard, retro-prospective studies on large cohorts of patients represent a unique tool to obtain reliable data on the natural history of these diseases (20).

The present study represents the first description of the clinical, immunologic and molecular features of a multi-centric cohort of patients affected with either typical or atypical SCIDs, followed within the IPINET network over a period of observation of 3 decades. In particular, the study was focused on presenting signs and natural history before HSCT or GT. As expected, the majority of the patients were diagnosed in the last decade of observation, thanks to improved awareness and broader availability of powerful genetic tests (16). In addition, in the last decade of observation, along with a progressive increase typical forms with very low AO, we also observed a significant number of the cases with a AO > 9 months. This may be explained by the recent increased awareness of atypical forms, that are often characterized by a later onset of the disease (21, 22). It is possible that the majority of these novel conditions were previously considered as “other not well-characterized” PIDs, or even misdiagnosed, since their presentation was often characterized by mild signs of immune dysfunction (23).

In a significant number of subjects (28% of the cohort), the SCID phenotype was part of a complex syndromic phenotype with multisystem anomalies, including multiple birth defects or neurological manifestations. Manifestations of immune dysregulation, mainly presenting as autoimmune cytopenia or ectodermal disorders, were observed more frequently in syndromic than in non-syndromic group (42 vs. 19%). Differently from what previously reported in other cohorts of syndromic SCID patients (24), we observed the same prevalence of severe infections in syndromic and non-syndromic patients. As expected, active and severe infections represent the most common cause of death in all the different categories of SCID patients. It is remarkable that 8% of children, previously considered “healthy,” presented with a single rapidly-progressive severe lung infection requiring ICU admission. Even if other groups have previously reported that 9% of all the PIDs patients require ICU admission during the follow-up (25), in this study we showed for the first time that admission to the ICU may prompt the suspicion of SCID. This observation should pave the way for future studies aimed at identifying the prevalence of SCID in children admitted in ICUs.

A peculiar observation in this cohort is the high proportion of patients with platelet count abnormalities, along with anemia, which were observed in almost half of the subjects. Differently from what expected, thrombocytosis was more frequent than thrombocytopenia and multivariate analysis demonstrated an interdependency of thrombocytosis with infections and inflammatory skin conditions as eczema.

Patients affected with DNA-RDs, typically, also show extra-hematopoietic alterations (26), which may predominate on clinical signs of immunodeficiency and lead to DD. In these subjects, the definition of the functional and molecular defect is essential for choosing the best therapeutic strategy. Due to the systemic nature of these disorders, the use of reduced intensity conditioning regimens is crucial to reduce mortality, systemic morbidity and long-term effects on other somatic cells harboring the genetic defect due to the effect of chemotherapy or ionizing radiation (27–30). The high mortality rate, observed by other groups, may be potentially explained either by the higher DD or by the increased DNA vulnerability (31, 32).

Genetic studies with traditional Sanger sequencing, HTS, or CGH-array were performed on 79% of the 111 patients. The overall diagnostic rate of the analyzed patients was 87.5%, and it was comparable to other cohorts (32, 33). No difference was observed in the rate of definitive genetic diagnosis among subjects of different geographic areas. Even though cytokine signaling defects were the most common group of genetic disorders in this study, the frequency of defects in genes encoding for proteins involved in the DNA recombination pathways (*RAG1, RAG2)* was similar to that of *IL2RG* gene defects, and accounted for the 26% of the whole cohort. This prevalence is higher than those recently reported in 2 prospective SCID studies performed in USA and in Asia (5, 34, 35). Although this observation may reflect a real difference in disease epidemiology, it should be noted that the rate of consanguineous marriages, can be higher in certain Italian geographic areas compared to other countries such as USA. Moreover, a founder effect may be a possible explanation for the high frequency of some specific mutations, such as the R225X mutation in *FOXN1* gene observed in the Acierno population (36).

In our study, SCIDs due to intrinsic thymic abnormalities, as cDGS, *FOXN1* defects and the recently described *PAX1* defect (36–38), accounted for 6% of the cases. This subgroup of SCIDs patients deserves careful consideration, since the traditional HSCT approach may be ineffective, or scarcely effective, and further therapies, as thymus transplantation should be considered (39–41).

Finally, the introduction into the clinical setting of the HTS technologies allowed the identification of a variety of very rare genetic forms, such as *TTC7A, IKBA, RFXANK, or CIITA* defects, that, otherwise would not have been, recognized. This observation further support a role for these technologies together with proper functional validation, as powerful diagnostic tool for PIDs.

Of note, about 25% of the subjects were not lymphopenic. It is noteworthy that OS/ME developed both in patients with normal/high age-matched lymphocytes count and in lymphopenic subjects, thus implying that also lymphopenic patients should be monitored for OS.

During the study period we identified a significant number of subjects with atypical forms, not fulfilling ESID diagnostic criteria. Among the group of atypical SCIDs with a known genetic cause, almost the totality of the subjects had an immunophenotype discordant to what expected on the basis of the genotype. In the group of subjects with *RAG1/2* defect, the presence of T cells was potentially explained by hypomorphic mutations and/or OS, or ME. Conversely, the atypical phenotype was associated with OS/ME only in 2 subjects with cytokine signaling defects. In particular, we observed that a significant proportion (about 40%) of *IL2RG-*mutated patients had near normal or low but detectable T-cell number. Hypomorphic mutations in several genes including *DCLRE1C, LIG4, RAG1*, and *JAK3* genes have been identified in patients with atypical SCID (42–46). To date, few patients harboring hypomorphic mutations in the *IL2RG* gene, responsible for an atypical phenotype, have also been described, the majority of them carrying the c.664C>T mutation (47, 48). In our group of *IL2RG* atypical SCID, 2 different hypormophic mutations, not previously reported in other case series, were identified in 2 unrelated families. Interestingly, one of them, harboring the p.E68K mutation, exhibited the coexistence of omenn-like disorder and ME, thus explaining the T^low^B^+^NK^+^ phenotype. In the remaining atypical *IL2RG* deficient patients, the mutations were severe, similarly to what previously reported by Mella et al. (49). In this latter group, further mechanisms, such as somatic cell gene reversion (50), mosaicisms, or a generic influence of environmental factors or of other gene modifiers may underlie the biological basis of the atypical phenotype. Interestingly, somatic mutations, can restore the wild-type sequence of the SCID-associated gene in a subset of lymphocytes, or introduce second-site mutations that permit the expression of partially functioning proteins (1). Similar hypotheses may also help to explain intrafamilial variability.

As for the two *JAK3-*mutated patients, they carried previously described hypomorphic mutations (46, 51). Functional immunological and genetic studies are mandatory to recognize such patients, since most of these patients had near normal total CD4 and/or CD8 counts.

Assuming a disease prevalence, based on the NBS available data, comprised between 1:40,000 and 1:58,000 (3), we would have expected about 285–415 cases of SCIDs over the 3 decades of observation (16,614,332 recorded live births). Thus, our data clearly indicate that, even though, it is possible that a few cases have been missed due to the voluntary participation to the study, the majority of the patients with SCID may have remained undiagnosed. In particular, we estimate that we have captured approximately only the 27–39% of all expected cases. However, a real estimate of the frequency of SCID in Italy is not possible. This clearly indicates that a nationwide NBS program is strictly necessary, and emphasizes the need to improve awareness of the broad phenotypic spectrum of SCID in order to facilitate diagnosis of cases with atypical signs of the disease (2).

## Data Availability

The raw data supporting the conclusions of this manuscript will be made available by the authors, without undue reservation, to any qualified researcher.

## Ethics Statement

The study was approved by the Coordinator Center Ethics Committee (Federico II Ethics Committee, registration number 61/17) and further approved by local Ethics Committees of all participating groups and providing informed consent. Informed and written consent had been obtained from the parents/legal guardians or from the participants when possible.

## Author Contributions

EC and CP collected, analyzed the data, and wrote the manuscript. CC, CA, SM, BM, APe, AT, SN, AF, RC, PP, ID'A, MP, AM, GG, RP, AS, GM, EA, SR, DM, FS, FB, MC, VL, SG, FL, PR, AA, RB, and APl collected the data and revised the manuscript. FE, FG, and GQ analyzed the data.

### Conflict of Interest Statement

The authors declare that the research was conducted in the absence of any commercial or financial relationships that could be construed as a potential conflict of interest.
