# Supplementary figures and images for: Clinical, Immunological, and Molecular Features of Typical and Atypical Severe Combined Immunodeficiency: Report of the Italian Primary Immunodeficiency Network
Source: Front Immunol. 2019 Aug 13;10:1908. doi: 10.3389/fimmu.2019.01908 (PMC6700292; doi:10.3389/fimmu.2019.01908)

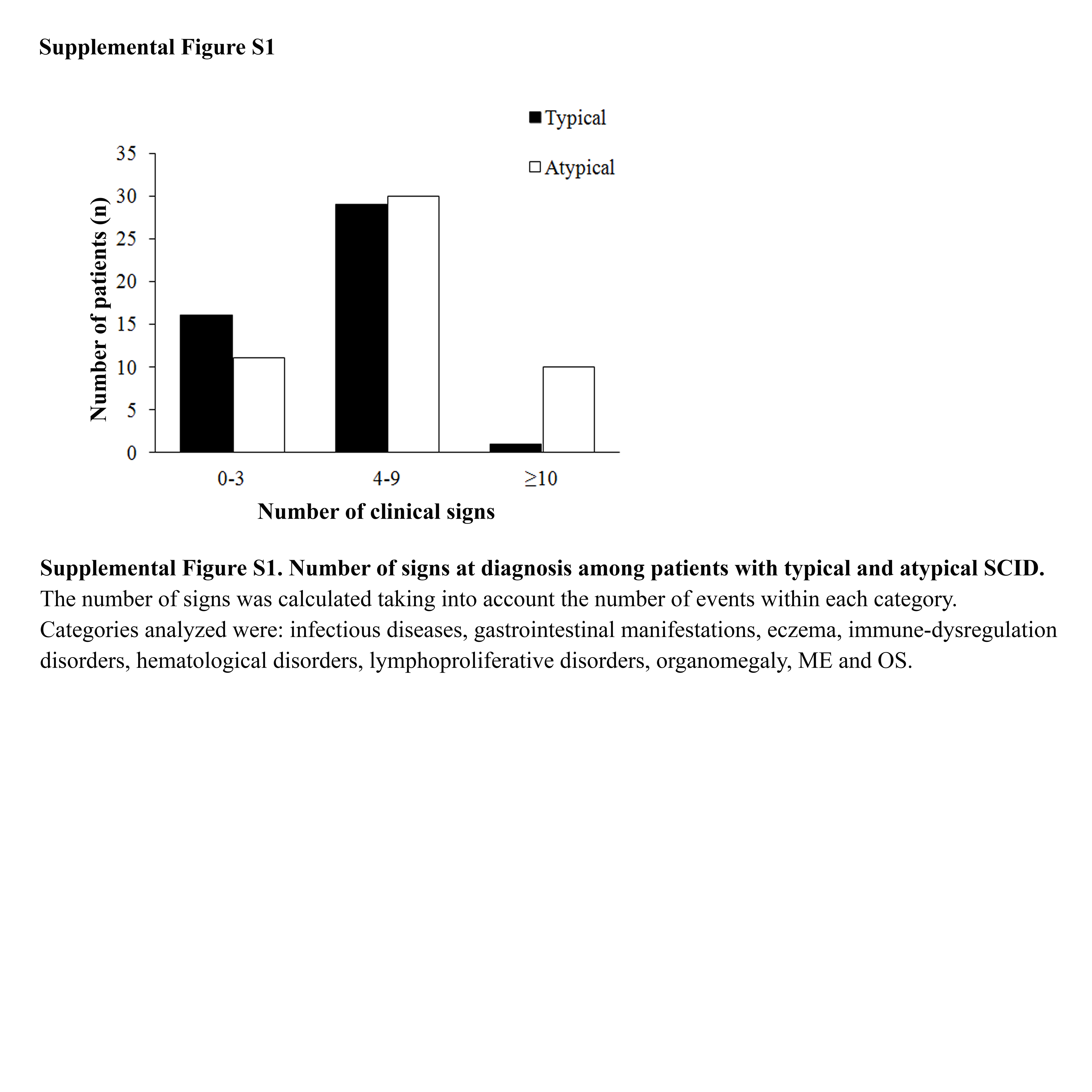

Supplement: Supplementary file 1 [file Image_1.JPEG]

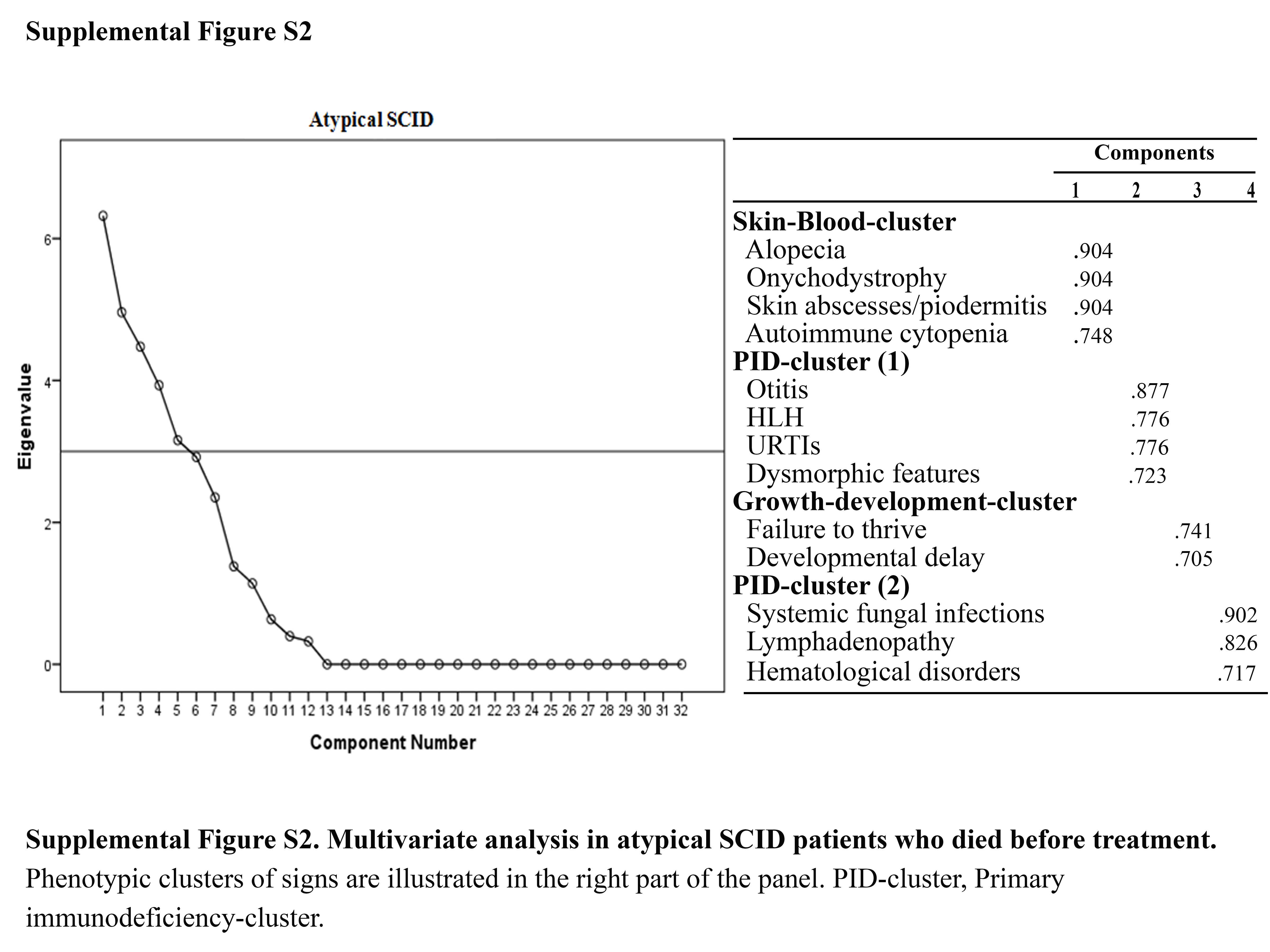

Supplement: Supplementary file 2 [file Image_2.JPEG]
