# Supplementary material for: Clinical, Immunological, and Molecular Features of Typical and Atypical Severe Combined Immunodeficiency: Report of the Italian Primary Immunodeficiency Network
Source: Front Immunol. 2019 Aug 13;10:1908. doi: 10.3389/fimmu.2019.01908 (PMC6700292; doi:10.3389/fimmu.2019.01908)
Supplement: Supplementary file 3 [file Table_1.docx]

**Supplemental Table S1**׀Clinical features of classical and atypical patients at diagnosis.

|  | **Overall (%)**  **No = 99** | **Typical (%)**  **No = 46** | **Atypical (%)**  **No = 53** |  |
| --- | --- | --- | --- | --- |
| **Feature** |  |  |  | ***P* value** |
| **Infections (N)** | 84 (84) | 41 (89) | 43 (81) | .205 |
| **Major respiratory tract infections** | 74 (75) | 36 (78) | 38 (72) | .453 |
| Pneumonia | 62 (63) | 35 (76) | 27 (51) | **.009** |
| Lobar | 23 (23) | 11 (24) | 11 (22) | .534 |
| Interstitial pneumopathy | 39 (35) | 24 (30) | 14 (48) | .123 |
| Lobar+Interstitial | 2 (2) | 0 (0) | 2 (4) | .497 |
| *P. Jiroveci* | 24 (61.5) | 14 (34) | 10 (19) | .180 |
| Unspecified | 15 (38.5) | 6 (13) | 9 (17) | .585 |
| Bronchiolitis | 17 (17) | 11 (24) | 6 (11) | .082 |
| Bronchiectasis | 3 (3) | 0 (0) | 3 (6) | .149 |
| **Other severe bacterial infections** | 19 (17) | 9 (11) | 10 (32) | **.023** |
| Sepsis | 10 (10) | 4 (9) | 6 (11) | .748 |
| Meningitis | 3 (3) | 0 (0) | 3 (10) | **.023** |
| Skin and lymph node abscesses | 4 (4) | 3 (4) | 1 (3) | 1.00 |
| Omphalitis | 1 (1) | 1 (2) | 0 (0) | .242 |
| Otitis | 11 (11) | 4 (9) | 7 (13) | .537 |
| **Systemic viral infections** | 31 (31) | 7 (14) | 24 (44) | **.001** |
| CMV | 17 (54) | 4 (9) | 13 (24.5) | **.037** |
| EBV | 7 (22.5) | 1 (2) | 6 (11) | .076 |
| HSV1/2 | 5 (16) | 1 (2) | 4 (7.5) | .223 |
| Adenovirus | 2 (6.5) | 1 (2) | 1 (2) | .919 |
| **Opportunistic infections** | 22 (22) | 14 (30) | 8 (15) | .067 |
| Candidiasis | 17 (17) | 11 (24) | 6 (11) | .082 |
| Disseminated fungal infection | 5 (5) | 3 (6.5) | 2 (4) | .433 |
| **BCG infection** | 5 (5) | 3 (6.5) | 2 (4) | 1.00 |
| *Mycobacterium tuberculosis* | 1(1) | 0 (0) | 1 (2) | .379 |
| **OS** | 18 (18) | 3 (6.7) | 15 (28) | **.008** |
| **ME**^*^ | 15 (28) | 7 (33) | 8 (25) | .986 |
| **Omenn-like +ME**^*^ | 3 (6) | 1 (5) | 2 (6) | .643 |
| **Gastrointestinal features** | 50 (50) | 22 (48) | 28 (53) | .689 |
| Chronic diarrhea | 23 (23) | 8 (17) | 15 (28) | .466 |
| Failure to thrive | 38 (38) | 18 (39) | 20 (38) | 1.00 |
| Villous atrophy | 6 (6) | 1 (1) | 5 (9) | **.026** |
| Protein loss entheropathy | 6 (6) | 0 (0) | 6 (11) | **.029** |
| **Eczema** | 26 (26) | 7 (15) | 19 (36) | **.019** |
| Transient or treatable | 4 (4) | 2 (4) | 2 (4) | .884 |
| Untreatable | 10 (10) | 4 (9) | 6 (11) | .665 |
| Erythrodermia | 12 (12) | 1 (2) | 11 (21) | **.004** |
| **Immune dysregulation** | 17(17) | 2 (4.3) | 15 (28) | **.001** |
| Autoimmune cytopenia | 12 (12) | 2 (4) | 10 (19) | **.026** |
| Granuloma | 3 (3) | 0 (0) | 3 (6) | .149 |
| Alopecia/ectodermal dystrophy | 5 (5) | 0 (0) | 5 (9) | .059 |
| Vasculitis | 2 (2) | 0 (0) | 2 (4) | .497 |
| Thyroiditis | 2 (2) | 0 (0) | 2 (4) | .497 |
| **Hematological disorders** | 16 (16) | 3 (6.5) | 13 (24.5) | .263 |
| HLH | 3 (3) | 0 (0) | 3 (5.7) | .149 |
| Lymphadenopathy | 13 (13) | 3 (6.5) | 10 (19) | .081 |
| Lymphoproliferative disorders | 2 (2) | 0 (0) | 2 (4) | .497 |
| **Organomegaly** | 26 (26) | 10 (22) | 16 (30) | .365 |
| Liver enlargement | 26 (26) | 10 (22) | 16 (30) | .369 |
| Spleen enlargement | 17 (17) | 5 (11) | 12 (23) | .181 |
| **Intensive care unit admission** | 9 (9) | 5 (11) | 4 (7.5) | .566 |

*CMV, cytomegalovirus; EBV, epstein barr virus; HSV, Herpes simplex virus; BCG, Bacillus Calmette Guerin; OS, Omenn syndrome;ME, Maternal Engraftment;HLH, Haemophagocytic Lymphohistiocytosis.*

*Bold indicates statistical significance (P < .05)*

***^*^Frequency has been calculated as percentage of patients tested for ME (n = 53; atypical n = 32)***
